# The *Glycine max* Conserved Oligomeric Golgi (COG) Complex Functions During a Defense Response to *Heterodera glycines*

**DOI:** 10.3389/fpls.2020.564495

**Published:** 2020-11-11

**Authors:** Bisho Ram Lawaju, Prakash Niraula, Gary W. Lawrence, Kathy S. Lawrence, Vincent P. Klink

**Affiliations:** ^1^Department of Biochemistry, Molecular Biology, Entomology and Plant Pathology, Mississippi State University, Starkville, MS, United States; ^2^Department of Biological Sciences, Mississippi State University, Starkville, MS, United States; ^3^Department of Entomology and Plant Pathology, Auburn University, Auburn, AL, United States; ^4^Center for Computational Sciences High Performance Computing Collaboratory, Mississippi State University, Starkville, MS, United States

**Keywords:** conserved oligomeric Golgi (COG) complex, soybean, nematode, disease resistance, harpin, elicitor, effector triggered immunity (ETI)

## Abstract

The conserved oligomeric Golgi (COG) complex, functioning in retrograde trafficking, is a universal structure present among eukaryotes that maintains the correct Golgi structure and function. The COG complex is composed of eight subunits coalescing into two sub-complexes. COGs1–4 compose Sub-complex A. COGs5–8 compose Sub-complex B. The observation that COG interacts with the syntaxins, suppressors of the erd2-deletion 5 (Sed5p), is noteworthy because Sed5p also interacts with Sec17p [alpha soluble NSF attachment protein (α-SNAP)]. The α-SNAP gene is located within the major *Heterodera glycines* [soybean cyst nematode (SCN)] resistance locus (*rhg1*) and functions in resistance. The study presented here provides a functional analysis of the *Glycine max* COG complex. The analysis has identified two paralogs of each COG gene. Functional transgenic studies demonstrate at least one paralog of each COG gene family functions in *G. max* during *H. glycines* resistance. Furthermore, treatment of *G. max* with the bacterial effector harpin, known to function in effector triggered immunity (ETI), leads to the induced transcription of at least one member of each COG gene family that has a role in *H. glycines* resistance. In some instances, altered COG gene expression changes the relative transcript abundance of syntaxin 31. These results indicate that the *G. max* COG complex functions through processes involving ETI leading to *H. glycines* resistance.

## Introduction

**FIGURE 1:**
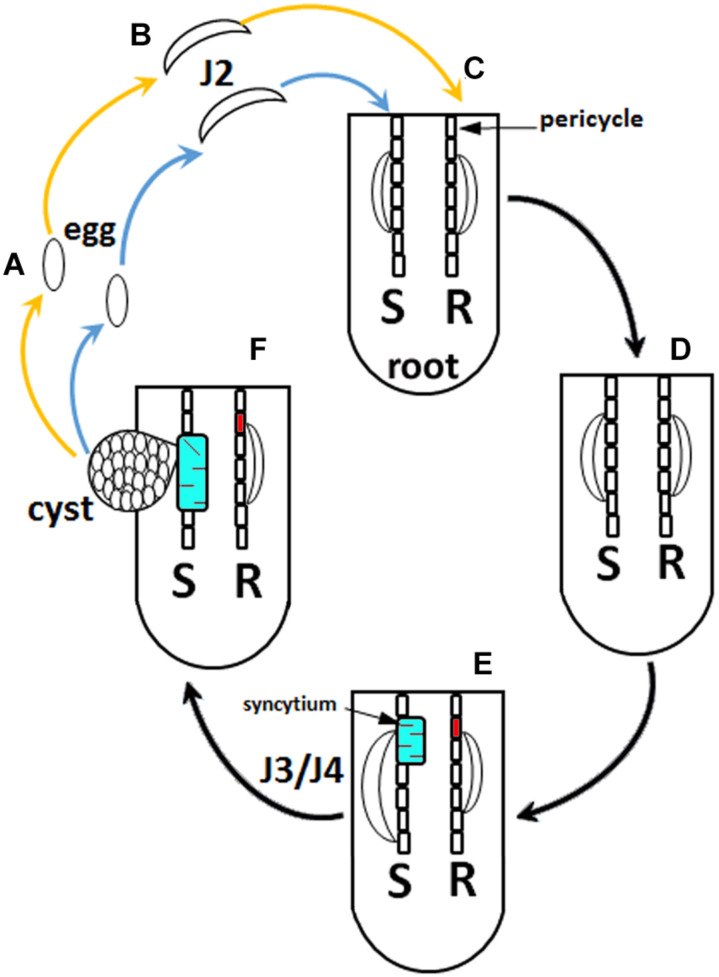
*Heterodera glycines* life cycle. **(A)** Eggs. **(B)** Second-stage juveniles (J2). **(C)** Root infected with J2 at 1 day post infection (dpi). **(D)** 3 dpi. **(E)** 6 dpi. **(F)** 30 dpi. The blue arrow indicates the nematode that will experience a compatible reaction, leading to plant susceptibility (S). The orange arrow indicates a nematode that will experience an incompatible reaction, leading to resistance (R). *H. glycines* development halts at the J2 stage during a resistant reaction in *G. max*_[Peking/PI 548402]_. Syncytia have been distinguished where, at 3 dpi, they are undergoing susceptible or resistant reactions that appear similar cytologically, showing features that include hypertrophy, an enlargement of nuclei, the development of dense cytoplasm, and an increase in endoplasmic reticulum (ER) and ribosome content. Due to these similarities, the 6 dpi time point is indicated, distinguishing a susceptible (red) and resistant (cyan) reaction because the syncytia undergoing a susceptible reaction are characterized by hypertrophy of nuclei and nucleoli, proliferation of cytoplasmic organelles, a reduction and dissolution of the vacuole, and cell expansion by incorporating adjacent cells. The resistant reaction, in contrast, shows cytoplasmic characteristics that are genotype-specific. The 6 dpi *G. max*_[Peking/PI 548402]_ reaction has cell wall appositions, structures that aggregate cytoplasmic components through actin polarization and vesicle-mediated delivery of cargo, the production of a necrotic layer of cells that surrounds the syncytium, and the accumulation of ER. The *G. max*_[Peking/PI 548402]_ resistant reaction leads to *H. glycines* development being blocked at the J2 stage. The *G. max*_[PI 88788]_ resistant reaction lacks cell wall appositions and lacks a necrotic layer of cells that surrounds the syncytium during the resistant reaction while having an accumulation of ER. The *G. max*_[PI 88788]_ resistant reaction leads to *H. glycines* development being blocked at J3–J4 stage.

*Heterodera glycines* [soybean cyst nematode (SCN)] is the most economically important pathogen of *Glycine max* (soybean). The infection of *G. max* by *H. glycines* accounts for a 7–10% decrease in yield and causes more economic loss than the rest of its pathogens combined losses (Wrather et al., 2001; Wrather and Koenning, 2006). *G. max* may show clear signs of *H. glycines* parasitism, including chlorosis and stunting. However, in some cases, no adverse signs of parasitism may be observed, except an approximately 15% decrease in yield (Wang et al., 2003). The life cycle of *H. glycines* is 30 or more days, dependent on ambient temperatures (Lauritis et al., 1983). During its obligate parasitic life cycle, *H. glycines* females become a hardened cyst, which is its carcass that contains 250–500 fertilized eggs. While within the cyst in the soil, the eggs may remain dormant for up to 9 years. Once the proper conditions occur, *H. glycines* eggs hatch as second-stage juveniles (J2s). Then, the J2s migrate toward and then burrow into the root. As they burrow, the J2s slice through root cells including epidermal, cortex, and endodermal cells with a mouth apparatus known as a stylet that is both rigid and tubular. It takes approximately 24 h for the J2 to reach its site of parasitism (Endo, 1965; Endo, 1991). Another function of the *H. glycines* stylet is to deliver effectors into a *G. max* pericycle or neighboring cell that it will parasitize. During this process, occurring over a period of days, the cell walls of the *H. glycines*-parasitized root cells dissolve. The cell walls dissolve through enzymatically driven processes facilitated by the nematode. As a result, 200–250 neighboring root cells are incorporated into a common cytoplasm producing a multinucleate syncytium. Notably, the syncytium is also where the localized defense response occurs, a process involving components of effector triggered immunity (ETI) and pathogen-activated molecular pattern (PAMP)-triggered immunity (PTI) (Ross, 1958; Endo, 1965, 1991; Jones and Dangl, 2006; Matsye et al., 2011, 2012; Pant et al., 2014; McNeece et al., 2017, 2019) (Figure 1).

Experiments performed in *G. max* show that part of its major resistance locus to the parasitic nematode *H. glycines*, *rhg1*, contains alpha soluble NSF attachment protein (α-SNAP) in tandemly copied arrays also including an amino acid transporter and a wound inducible protein (Caldwell et al., 1960; Matsye et al., 2011; Cook et al., 2012). Subsequent experiments have shown α-SNAP has a role in resistance (Matsye et al., 2012; Cook et al., 2012; Sharma et al., 2016). However, the genetic diversity of *H. glycines* has both complicated and facilitated the analysis of the *rhg1* locus and resistance in general (Golden et al., 1970; Riggs and Schmitt, 1988). These observations are consistent with experiments showing membrane fusion apparatus proteins participating in vesicle transport having a role in the plant defense to pathogens (Collins et al., 2003). In those experiments, the SNARE protein syntaxin 121 (SYP121) functions in impairing fungal haustorial penetration, and thus, the mutant locus was first called *penetration1* (*pen1*) (Collins et al., 2003). Since those initial observations, numerous other membrane fusion proteins found at various subcellular membrane-enveloped structures have been shown to function in defense processes (Lipka et al., 2005). More recent experiments performed in *Hordeum vulgare* (wheat) identified its COG3 (*Hv*COG3) functions during its defense response to cellular penetration by the fungal pathogen *Blumeria graminis* f.sp. hordei. (Ostertag et al., 2013). A number of *G. max* homologs of membrane fusion proteins, including 20S particle components and myosin XI, have been shown to function during the resistant reaction it has to parasitic nematodes (Matsye et al., 2012; Sharma et al., 2016; Klink et al., 2017; Austin et al., 2019). However, the COG complex has not yet been studied.

The α-SNAP gene was originally identified in *Saccharomyces cerevisiae* as Sec17 in a genetic screen designed to find proteins that function in secretion (Novick et al., 1980). The α-SNAP gene is part of a larger complex known as the 20S particle that functions in the fusion of vesicles to target membranes, leading to the secretion of cargo (Söllner et al., 1993a,b). Experiments performed in *S. cerevisiae* show Sec17p binds to the suppressors of the erd2-deletion 5 (Sed5p), known in plants as syntaxin 31 (Hardwick and Pelham, 1992; Lupashin et al., 1997; Bubeck et al., 2008). Both α-SNAP and syntaxin 31 have roles in the defense process *G. max* has to *H. glycines* (Matsye et al., 2012; Cook et al., 2012; Pant et al., 2014; Sharma et al., 2016). Sed5p is involved in retrograde trafficking through its interaction with the conserved oligomeric Golgi (COG) complex (Shestakova et al., 2007). Therefore, an indirect link exists between COG and α-SNAP through Sed5p and implicates the COG complex as having an important role in the defense process that *G. max* has toward *H. glycines* parasitism.

The COG complex is universal among eukaryotes, maintaining the correct structure and function of the Golgi apparatus (Ungar et al., 2002; Vukašinović and Žárský, 2016). An important facet of the COG complex is its role in retrograde trafficking occurring between the Golgi cisternae (Ungar et al., 2002). Furthermore, as part of its function, the COG complex plays an important role in the homeostasis of enzyme glycosylation (Ungar et al., 2002).

The COG complex is composed of eight subunits (Whyte and Munro, 2001; Ungar et al., 2002; Willett et al., 2013). The eight COG complex subunits coalesce into two sub-complexes (Fotso et al., 2005; Ungar et al., 2005). Sub-complex A is composed of COGs1–4, while sub-complex B is composed of COGs5–8 (Ungar et al., 2002). Components of the COG complex interact with the soluble *N*-ethylmaleimide-sensitive factor attachment protein receptor (SNARE), which is part of the 20S particle, to effect membrane fusion (Söllner et al., 1993a,b; Cottam and Ungar, 2012; Jahn and Fasshauer, 2012; Willett et al., 2013). The COG complex also functions along with other SNARE interacting proteins, Rabs, tethers containing coiled-coil proteins and various molecular motors to perform its functions (Cottam and Ungar, 2012; Willett et al., 2013). Consequently, the COG complex performs a central role in the retrograde movement of materials between Golgi cisternae.

Much understanding of the COG complex function has come from mutant analyses in *S. cerevisiae*. For example, *S. cerevisiae* mutants of COGs1–3 (i.e., *sec36*, *sec35*, and *sec34*, respectively) exhibit very slow growth, while COG4 (*cod1*/*sec38*) mutants are inviable (Whyte and Munro, 2001; Ram et al., 2002). In contrast, mutants of COGs5–8 (*cod4*, *sec37*, *cod6*, and *dor1*, respectively) are viable. The growth deficiencies are caused by various impairments of Golgi function involving retrograde trafficking.

In plants, the *Arabidopsis thaliana embryo yellow* (*eye*) mutant phenotype, which has impaired cell expansion and meristem organization, results from a mutation in the AtCOG7 gene AT5G51430 (Ishikawa et al., 2008). A surprising observation in *A. thaliana* is that AtCOG2 (AT4G24840) performs a role in recruiting the exocyst complex to xylem cell sites of secondary cell wall deposition (Oda et al., 2015). This process appears to involve the targeting of the exocyst to microtubules through the direct interaction of exocyst subunits with the COG2 protein (Vukašinović et al., 2017). COG3 (AT1G73430) and COG8 (AT5G11980) perform a number of functions including modulating the morphology of the Golgi apparatus and homeostasis during vesicle trafficking and also being essential for pollen tube growth (Tan et al., 2016). COG6 (AT1G31780) is essential for the maintenance of the Golgi structure and pollen tube growth (Rui et al., 2020). No clear role has been shown for *A. thaliana* COG1 (AT5G16300), COG4 (AT4G01400), or COG5 (AT1G67930). For a detailed treatise on the plant COG complex and the plant vesicle transport system in general, the reviewer is directed to Vukašinović and Žárský (2016). Consequently, in contrast to what is understood in *S. cerevisiae*, very little is known about the COG complex in plants and even less regarding its role during pathogenic interactions. Therefore, the interaction between *G. max* and *H. glycines* presents an opportunity to better understand plant defense responses, especially since α-SNAP and a COG-interacting protein, syntaxin, function in the resistant reaction (Cook et al., 2012; Matsye et al., 2012; Pant et al., 2014).

The study presented here is devoted to the characterization of the entire COG complex gene family of *G. max* as it relates to determining a potential role during its resistant reaction to the parasitic nematode *H. glycines*. The experiments presented here began by determining whether the COG complex gene family members are present in the genome of *G. max*. The 16 *G. max* COG complex genes have been cloned and genetically engineered for their overexpression in the *H. glycines*-susceptible genotype *G. max*_[Williams 82/PI 518671]_ to see if that susceptible genotype became resistant to parasitism. In contrast, the entire COG complex gene family has been cloned and genetically engineered for its suppressed expression by RNA interference (RNAi) in the *H. glycines*-resistant genotype *G. max*_[*Peking/PI* 548402]_ to see if that resistant genotype became susceptible to parasitism. COG gene expression assays using reverse transcriptase quantitative PCR (RT-qPCR) demonstrate the appropriate increased or decreased transcript abundances of the COG complex genes as a consequence of their transgenically driven overexpression or RNAi, respectively. The experiments demonstrate the involvement of specific COG complex gene family members functioning during the engineered resistance reaction, while their suppressed transcription by RNAi leads to engineered susceptibility to *H. glycines* parasitism. Furthermore, treatment of *G. max* with the bacterial elicitor harpin leads to induced expression of COG genes that function in resistance to *H. glycines*. This result indicates that ETI plays a role in activating the COG gene expression that functions in *G. max* resistance to *H. glycines*. Lastly, in some cases, COG gene expression appears to regulate the expression of syntaxin 31.

## Materials and Methods

### Experiment Details

Three independent biological replicates have been used for each experiment with each independent biological replicate having 10–25 experimental replicate plants. For RT-qPCR studies, three independent biological replicate lines were compared. For the female index (FI) studies using transgenic COG-overexpressing (OE), COG-RNAi, and their respective pRAP15-*ccd*B and pRAP17-*ccd*B control lines, the analysis used three independent biological replicates, each independent biological replicate having 10–25 experimental replicate plants. For the harpin effector RT-qPCR analyses, three independent biological replicates have been used, employing the same RNA that had been used in Aljaafri et al. (2017). A total of 10 plants were studied in each biological replicate and control in the experiments of Aljaafri et al. (2017). For the syntaxin 31 RT-qPCR analyses, three independent biological replicates have been used. Consequently, three independent biological replicates have been used in these analyses. Furthermore, a minimum of three independent biological replicates have been used in order to enable an assessment of significance.

### Conserved Oligomeric Golgi Gene Identification

The *G. max* genome sequence, assembly, and annotation are housed at Phytozome^1^ (Goodstein et al., 2012). The *G. max* proteome has been queried with the conceptually translated *A. thaliana* COG gene sequences using the Basic Local Alignment Search Tool (BLAST) (Altschul et al., 1990). The comparative analyses have been performed in Phytozome using the default settings, including Target type: Proteome; Program: BLASTP-protein query to protein database; Expect (E) threshold: −1; Comparison matrix: BLOSUM62; Word (W) length: default = 3; number of alignments to show: 100 allowing for gaps and filter query.

### Gene Cloning and Generation of Transgenic *G. max*

The more recent *Glycine max* Wm82.a2.v1 annotation has been used in the design of PCR primer sequences (Supplementary Table 1) (Lawaju et al., 2018). COG gene amplicons are generated by PCR using the Accuprime Taq Polymerase System (Invitrogen) according to the manufacturer’s instructions. The PCR reaction contents are run on a 1% agarose gel with the COG gene amplicons purified using the Wizard SV Gel and PCR Clean-Up System (Promega) according to the manufacturer’s instructions. Cloning of the COG amplicons is accomplished through ligation into the pENTR/D-TOPO entry vector using the pENTR/D-TOPO Cloning Kit (Invitrogen) according to the manufacturer’s instructions. Subsequently, the ligation reaction contents undergo transformation into One Shot TOP10 Chemically Competent *Escherichia coli* (TOP 10) (Invitrogen) cells according to the manufacturer’s instructions. Chemical selection occurs on Luria–Bertani (LB) agar plates that contain 50 μg/ml kanamycin. After 14 h, colonies are picked and grown in 3 ml of liquid LB containing 50 μg/ml kanamycin. After 14 h, the colonies undergo plasmid isolation using the Wizard *Plus* SV Minipreps DNA Purification System (Promega) according to the manufacturer’s instructions. The confirmed COG gene amplicons are ligated into the Gateway-compatible overexpression (pRAP15) or RNAi (pRAP17) destination vectors using the Gateway LR Clonase Enzyme mix (Invitrogen) according to their instructions (Curtis and Grossniklaus, 2003; Klink et al., 2009; Matsye et al., 2012). The promoter driving the expression of the overexpression and RNAi cassettes is the figwort mosaic virus (FMV) sub-genomic transcript (Sgt) promoter (Bhattacharyya et al., 2002). The FMV-Sgt promoter consists of a 301-bp FMV Sgt promoter fragment [sequence −270 to +31 from the transcription start site (TSS)] (Bhattacharyya et al., 2002). The promoter has been used in the design of the pRAP15 and pRAP17 vectors (Klink et al., 2009; Matsye et al., 2012). The RNAi cassette of pRAP17 is designed to produce a hairpin RNA having inverted repeats (Curtis and Grossniklaus, 2003; Klink et al., 2009). The experimental controls are the un-engineered pRAP15 or pRAP17 vectors. However, these vectors have the *ccd*B gene that is positioned where, otherwise, the COG gene amplicon is directionally inserted during the LR clonase reaction. Consequently, this feature makes the un-engineered vectors [pRAP15-*ccd*B (overexpression control) and pRAP17-*ccd*B (RNAi control)] suitable as controls for any non-specific effects from gene overexpression or RNAi (McNeece et al., 2019). The LR reaction contents undergo transformation into chemically competent *E. coli* TOP 10 cells, having been performed according to the manufacturer’s instructions. Chemical selection occurs on LB agar plates that contain 5 μg/ml tetracycline. COG gene-specific primers are used in PCR reactions to confirm the presence of the targeted gene (Supplementary Table 1). The COG gene-containing pRAP15/17 destination vectors undergo transformation into chemically competent *Agrobacterium rhizogenes* K599 (K599) using the freeze–thaw transformation procedure (Hofgen and Willmitzer, 1988; Haas et al., 1995; Collier et al., 2005; Lawaju et al., 2018).

### Production of Transgenic Plants for Functional Experiments

Transgenic plants have been generated according to Lawaju et al. (2018). The 250-ml culture of K599 that is transformed with the COG-containing plasmid is pelleted through centrifugation in a Sorvall RC6 Plus Superspeed Centrifuge at 4^*o*^C for 20 min. The resulting K599 pellet is resuspended in Murashige and Skoog medium including vitamins (MS) (Duchefa Biochemie) containing 3.0% sucrose, pH 5.7 (MS media). The COG genes have been engineered to undergo overexpression in the *H. glycines*-susceptible genotype *G. max*_[Williams 82/PI 518671]_. In contrast, the COG genes have been engineered to undergo RNAi in the *H. glycines*-resistant genotype *G. max*_[Peking/PI 548402]_. Transgenic *G. max* plants are made by cutting off the root of a 1-week-old plant at the hypocotyl using a new, sterile razor blade. The plants are immersed in the K599 solution in a Petri dish prior to cutting. This step provides the transformed K599 immediate access into the wound site made by the removal of the root. Then, 25 rootless plants are placed in a 140-ml glass beaker containing 25 ml of transformed K599 in MS media solution. Infiltration of the plant tissue, having the transformed K599, occurs under a vacuum for 5 min. The pump is turned off after 5 min, and the plants are left in the vacuum for an additional 10 min. The vacuum is then slowly released, allowing the transformed K599 further entry into the plant tissue. After the vacuum is completely released, the cut ends of *G. max* are placed individually into fresh coarse Vermiculite Grade A-3 (Palmetto Vermiculite) in 50-cell 725602C Propagation Trays held in 710245C Standard Flats with holes in the bottom (T.O. Plastics) at a depth of 3–4 cm deep. The plant trays are then placed in a Sterilite, 25 Qt/23 L Modular Latch Box (Sterilite). The container is then covered with its lid. The covered containers are placed under lights at a distance of 20 cm from standard fluorescent cool white 4,100 K, 32-watt bulbs. The bulbs, emitting 2,800 lumens (Sylvania), are used to illuminate the plants for 5 days at ambient lab temperatures (22°C) on a 16/8 light/dark cycle. The plants are subsequently transferred to the greenhouse. The plants are removed from the container, allowing their recovery for 1 week. Visual selection of transgenic *G. max* roots occurs with the enhanced green fluorescent protein (eGFP) visual reporter (Haseloff et al., 1997). Visualization occurs using the Dark Reader Spot Lamp (SL10S) (Clare Chemical Research). Roots exhibiting the eGFP reporter expression will also possess the COG gene expression cassette. The eGFP and COG genes each have their own promoter and terminator sequences. COG gene transfer occurs because K599 shuttles the DNA cassette located between the left and right borders of the pRAP15 and pRAP17 destination vectors into the root cell chromosomal DNA. As a consequence, the gene is stably integrated into the root somatic cell chromosome, even though the DNA cassette is not incorporated into the germline (Tepfer, 1984; Haas et al., 1995; Collier et al., 2005). The resultant root develops from the base of a non-transgenic shoot stock, leading to the production of a genetically mosaic plant. Therefore, each individual transgenic root system is an independent transformant line. Culture of the transgenic plants occurs in Ray Leach “Cone-tainer” (SC10) pots (Stuewe and Sons, Inc.). These pots are secured in a Ray Leach Tray (RL98) (Stuewe and Sons, Inc.). The soil is a sandy (93.00% sand, 5.75% silt, and 1.25% clay) mixture. The plants are then allowed to recover for 2 weeks prior to the start of the experiments. The effect that the genetic constructs have on *G. max* COG gene expression is confirmed by RT-qPCR. (Please refer to RT-qPCR-related section.)

### PCR and Reverse Transcriptase Quantitative PCR of Conserved Oligomeric Golgi Complex Gene Family Members

The determination of the extent of COG gene expression in transgenic *G. max* is accomplished by RT-qPCR according to Lawaju et al. (2018). The analysis procedure uses Taqman 6-carboxyfluorescein (6-FAM)-labeled probes and Black Hole Quencher (BHQ1) (MWG Operon) (Supplementary Table 1) according to the manufacturer’s instructions. The control used in the RT-qPCR experiments is designed from a ribosomal S21 (RPS21) protein coding gene (Glyma.15G147700) (Supplementary Table 1). The relative change in gene expression that is caused by the genetic engineering event is calculated using 2^–ΔΔ*CT*^ (Livak and Schmittgen, 2001). The p-values have been calculated using a Student’s *t*-test for the replicated RT-qPCR reactions (Yuan et al., 2006). Experiments and statistical analyses have been performed from three independent biological replicates.

### Assaying the Effect Altered Conserved Oligomeric Golgi Gene Expression Has on Nematode Parasitism

*Heterodera glycines* infections of transgenic plants are performed according to the procedures described in Lawaju et al. (2018). *H. glycines*_[NL1–Rhg/HG–type 7/race 3]_ eggs are obtained from cysts, collected from 60-day-old greenhouse-grown *G. max* stock plants grown in 500-cm^3^ polystyrene pots. Extraction of the *H. glycines* cysts from the stock *G. max* plants occurs by sucrose flotation. The roots containing the *H. glycines* cysts are then washed. Washing occurs through nested 850-μm-pore and 250-μm-pore sieves. Collection of the *H. glycines* cysts occurs from the 250-μm-pore sieve. A mortar and pestle are used to grind the *H. glycines* cysts to release eggs. Gravitational sieving and subsequent sucrose centrifugation are used to obtain the *H. glycines* eggs. Nested 75-μm-pore over 25-μm-pore sieves are used to recover the *H. glycines* eggs. Subsequently, *H. glycines* J2s are collected from hatched eggs. This occurs using a modified Baermann funnel that is placed on a Slide Warmer (Model 77) (Marshall Scientific) at 28°C. Hatching *H. glycines* egg occurs after 4–7 days. Subsequently, *H. glycines* J2s are collected. Collection occurs on a 25-μm-pore sieve, placed in 1.5-ml tubes. Centrifugation of the tube and its contents occurs at 10,000 rpm for 1 min. This step is followed by washing the contents with sterile distilled water and subsequent centrifugation again. Concentrating the J2s occurs by centrifugation in an IEC clinical centrifuge. This step is done for 30 s at 1,720 rpm to obtain a final optimized concentration of 2,000 pi-J2/ml. Subsequently, each plant having the transgenic root is inoculated with 1 ml of *H. glycines*. The *H. glycines* concentration is 2,000 J2s/ml per root system (per plant). Infection of the transgenic root systems by *H. glycines* is allowed to proceed for 30 days. At the experiment’s conclusion, the cysts are collected over nested 20- and 100-mesh sieves. To ensure collection of all cysts for enumeration of the female index (FI), the soil is washed several times and the rinse water sieved.

The community-accepted standard representation of the obtained data is the FI; FI = (Nx/Ns) × 100 (Golden et al., 1970). In the experiments presented here, Nx is the pRAP-COG gene-transformed (experimental) line. Ns is the pRAP-ccdB (control) line. For the analysis purposes presented here, the FI is calculated as cysts per whole root system (wr) grown within 100 cc of soil and also cysts per gram (pg) of root system. Each analysis has its own purpose. From a historical perspective, the wr system analysis determines the FI regardless of any differences in *G. max* or *H. glycines* genotype regarding root performance prior to or during infection. In contrast, the cyst pg of root system analysis accounts for possible altered root growth that is being caused by the influence of the overexpression or RNAi of the candidate *G. max* COG gene and/or nematode. For every experiment, three biological replicates are made for each construct. Furthermore, each biological replicate has 10–25 individual transgenic plants each that serve as experimental replicates. The pRAP15-*ccd*B overexpression control had 943.09 ± 275.47 cysts per root system in 100 cc of soil (*p* < 0.001). The pRAP17-ccdB RNAi had 7.77 ± 2.29 cysts per root system in 100 cc of soil (*p* < 0.001). Statistical analyses have employed the Mann–Whitney–Wilcoxon (MWW) rank-sum test, which is a non-parametric test of the null hypothesis not requiring the assumption of normal distributions (*p* < 0.05 cutoff) (Mann and Whitney, 1947). Root mass has been determined from fresh weight and analyzed using MWW (*p* < 0.05 cutoff). Experiments and statistical analyses have been performed from three independent biological replicates, with each biological replicate having 15–25 individual transgenic plants.

## Results

### Identification of *G. max* Conserved Oligomeric Golgi Complex Genes and Expression

The purpose of the analysis presented here is to understand whether, in *G. max*, its COG complex functions during the resistant reaction to *H. glycines* parasitism. Protein sequences of the eight COG complex subunits have been identified in *A. thaliana* (Table 1). The *A. thaliana* protein sequences have been used to query the proteome of *G. max* using protein BLAST analyses. The outcome of those analyses is the identification of the *G. max* COG complex homologs (Table 1).

**TABLE 1:** Conserved oligomeric Golgi (COG) complex gene information.

*Arabidopsis thaliana*	*Glycine max*				

Gene	Accession Gene	Gene	Wm82.a1.v1.1	Wm82.a2.v1	Percent ID
COG1	AT5G16300	COG1-1	Glyma10g34570	Glyma.10G201900	62%
		COG1-2	Glyma20g32980	Glyma.20G188500	63%
COG2	AT4G24840	COG2-1	Glyma17g13840	Glyma.17G129100	70%
		COG2-2	Glyma05g03260	Glyma.05G047300	68%
COG3	AT1G73430	COG3-1	Glyma13g17521	Glyma.13G114900	80%
		COG3-2	Glyma17g04990	Glyma.17G045100	79%
COG4	AT4G01400	COG4-1	Glyma19g44940	Glyma.19G260100	79%
		COG4-2	Glyma03g42200	Glyma.03G261100	78%
COG5	AT1G67930	COG5-1	Glyma14g03340	Glyma.14G029500	73%
		COG5-2	Glyma02g45580	Glyma.02G286300	73%
COG6	AT1G31780	COG6-1	Glyma01g35820	Glyma.01G154500	66%
		COG6-2	Glyma11g09550	Glyma.11G090100	66%
COG7	AIU51104	COG7-1	Glyma09g35790	Glyma.09G224000	78%
		COG7-2	Glyma12g01570	Glyma.12G013000	78%
COG8	AT5G11980	COG8-1	Glyma16g22880	Glyma.16G120600	78%
		COG8-2	Glyma02g04920	Glyma.02G043400	80%

### Functional Analysis of the Conserved Oligomeric Golgi Complex Gene Family

**FIGURE 2:**
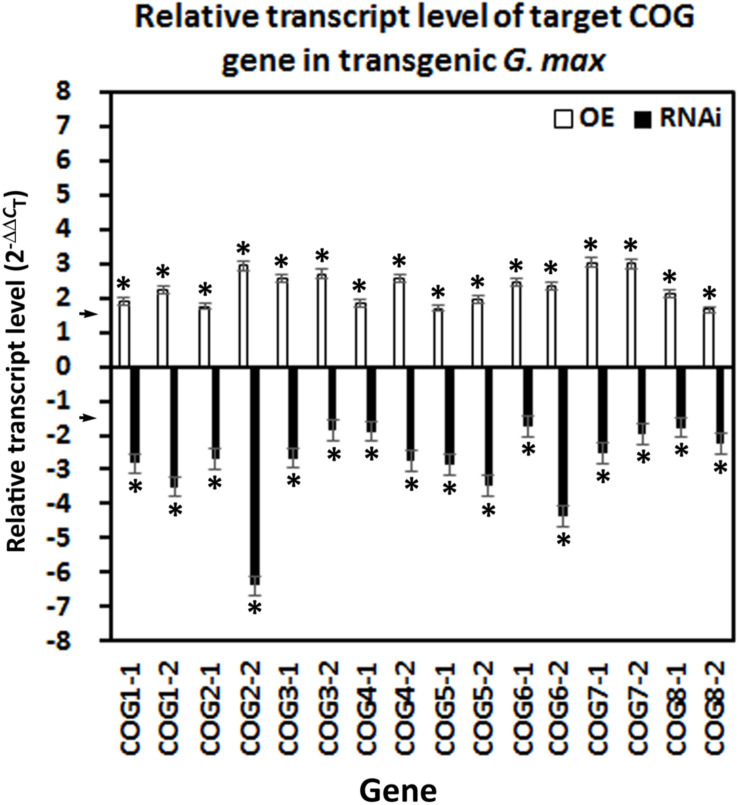
Confirmation of conserved oligomeric Golgi (COG) overexpressing (OE) and RNAi effect by RT-qPCR. In each transgenic line, gene expression has been altered accordingly with the overexpression lines increasing transcript abundance by ≥1.5-fold using the (2^–ΔΔ^*^*C*^*_*T*_) method (Livak and Schmittgen, 2001) (*p* < 0.05, Student’s *t*-test) and the RNAi lines by ≤1.5-fold (*p* < 0.05, Student’s *t*-test). The arrows on the *y*-axis indicate the level where the experimental outcome is statistically significant [*p* < 0.05, Mann–Whitney–Wilcoxon (MWW)]. Experiments and statistical analyses have been performed from three independent biological replicates. Statistically significant *p* < 0.05.

The overexpression of the 16 COG genes has been accomplished in the *H. glycines*-susceptible *G. max*_[Williams 82/PI 518671]_, along with the appropriate pRAP15-*ccd*B control. In contrast, RNAi of the 16 COG genes has been accomplished in the *H. glycines*-resistant *G. max*_[Peking/PI 548402]_, along with the appropriate pRAP17-*ccd*B control. RNA has been isolated from the transgenic lines and converted to cDNA for its use in RT-qPCR experiments. As expected, the overexpression of the COG genes leads to an increase in their relative transcript abundance as compared to their pRAP15-*ccd*B root tissue control when using the RPS21 gene as a gene expression control (*p* < 0.05, Student’s *t*-test) (Figure 2). In contrast, RNAi of the COG genes leads to a decrease in their relative transcript abundance as compared to their pRAP17-*ccd*B root tissue control when using the RPS21 gene as a gene expression control (*p* < 0.05, Student’s *t*-test) (Figure 2). Consequently, the roots are functioning as expected regarding the relative transcript abundance of the COG genes. Therefore, the roots could be used for functional experiments to determine the effect the gene cassette has on *H. glycines* parasitism.

### Functional Experiments Examine the Conserved Oligomeric Golgi Gene Role During Resistance to *H. glycines* Parasitism

**FIGURE 3:**
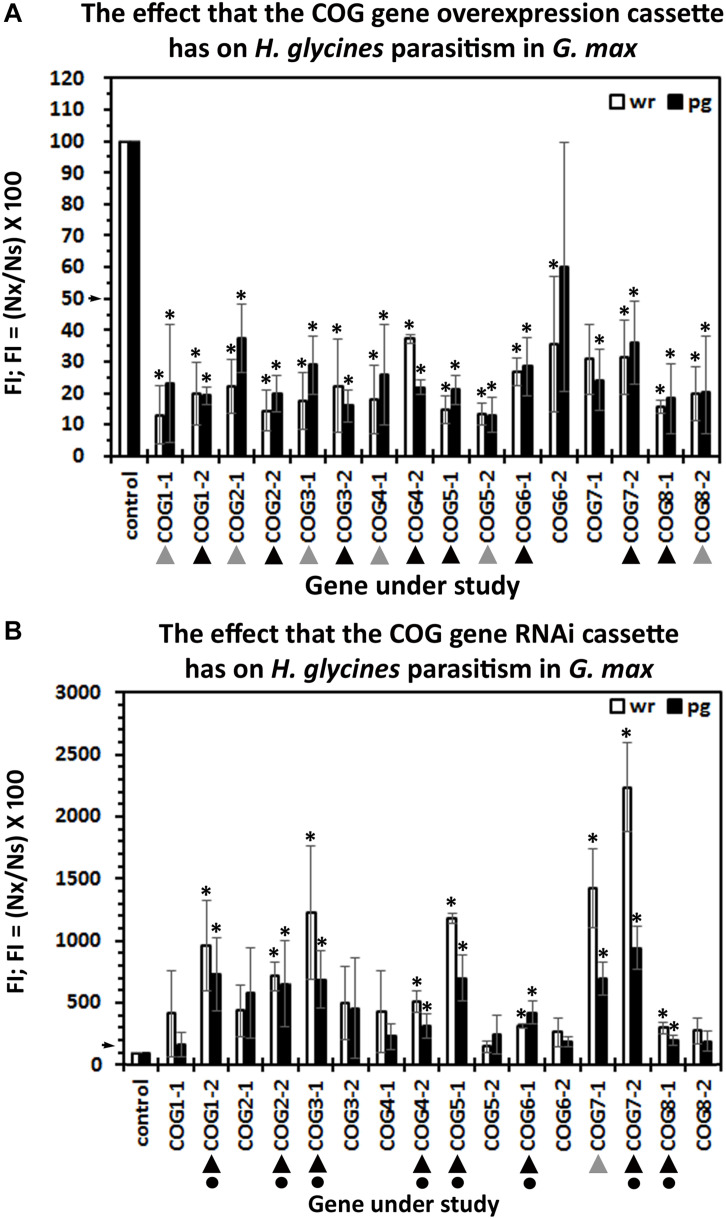
The female index (FI) of transgenic *G. max*. **(A)** Overexpression: roots engineered to increase the relative transcript abundance of the target conserved oligomeric Golgi (COG) gene through overexpression. **(B)** RNAi: The FI of transgenic *G. max* engineered to decrease the relative transcript abundance of the target COG gene; wr, whole root analysis; per gram, per gram analysis. Statistically significant (*p* < 0.05, Student’s *t*-test). The pRAP15-ccdB overexpression control had 943.09 ± 275.47 cysts per root system in 100 cc of soil (*p* < 0.001). The pRAP17-ccdB RNAi had 7.77 ± 2.29 cysts per root system in 100 cc of soil (*p* < 0.001). (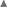) Decreases *H. glycines* parasitism in the overexpressing (OE) lines or increases parasitism in the RNAi lines, but not both. Decreases *H. glycines* parasitism in the OE lines and increases parasitism in the RNAi lines (▲), and therefore, the gene is considered functioning in defense (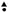) because it meets the criteria of suppressing parasitism by 50% in the overexpression lines and increasing parasitism in the RNAi lines by 1.5-fold (*p* < 0.05, MWW). The arrows on the *y*-axis indicate the level where the experimental outcome is statistically significant (*p* < 0.05, MWW). Experiments and statistical analyses have been performed from three independent biological replicates each having 10–25 independent transgenic root systems. Statistically significant *p* < 0.05.

The number of experimental replicates (10–25 plants) within each biological replicate (*n* = 3) is provided (Supplementary Table 2). The soil containing the potted transgenic plants has been infested with 2,000 *H. glycines* J2s. Infection of the roots has been permitted to proceed for 30 days with subsequent extraction of cysts. Enumeration of cysts from the pRAP15-*ccd*B control and COG-overexpressing experimental lines has been performed. The analysis has resulted in the calculation of the FI from the overexpression lines for both cysts per wr system and cysts pg of wr system (Figure 3). The results of the analysis show that the overexpression of 14 of the 16 COG genes suppresses *H. glycines* parasitism by 50% or greater in the *H. glycines*-susceptible *G. max*_[Williams 82/PI 518671]_ as compared to the pRAP15-*ccd*B control (*p* < 0.05, MWW). The only COG overexpression lines that did not have a statistically significant effect on *H. glycines* parasitism as compared to the pRAP15-*ccd*B control are COG6-2-OE and COG7-1-OE (*p* ≥ 0.05, MWW). These experiments are complimented by RNAi of each COG gene in the *G. max*_[Peking/PI 548402]_ genotype, which is normally *H. glycines*-resistant. The enumeration of cysts from pRAP17-*ccd*B control and COG-RNAi experimental lines has been made in *G. max*_[Peking/PI 548402]_, resulting in the calculation of the FI for both cysts per wr system and cysts pg of wr system (Figure 3). The results of the analysis show that the RNAi of nine COG genes, including COG1-2, COG2-2, COG3-1, COG4-2, COG5-1, COG6-1, COG7-1, COG7-2, and COG8-1, increases *H. glycines* parasitism by 1.5-fold (FI ≥ 150) or greater (*p* < 0.05, MWW) (Figure 3). In contrast, RNAi of COG1-1, COG2-1, COG3-2, COG4-1, COG5-2, COG6-2, and COG8-2 does not increase *H. glycines* parasitism in the *H. glycines*-resistant *G. max*_[Peking/PI 548402]_ by our parameters (*p* ≥ 0.05, MWW). To satisfy our criteria of a COG gene functioning in resistance, a statistically significant decrease in *H. glycines* parasitism as determined by the FI result must be obtained in each overexpression replicate in both the cyst per wr system and cyst pg of wr system analyses and in each RNAi replicate in both the cyst per wr system and cyst pg of wr system analyses (*p* < 0.05, MWW). The results presented here show that those criteria have been met only for eight of the COG genes including COG1-2, COG2-2, COG3-1, COG4-2, COG5-1, COG6-1, COG7-2, and COG8-1 (Figure 3). Consequently, at least one paralog of each COG gene family satisfies our criteria as functioning in resistance to *H. glycines* parasitism.

**FIGURE 4:**
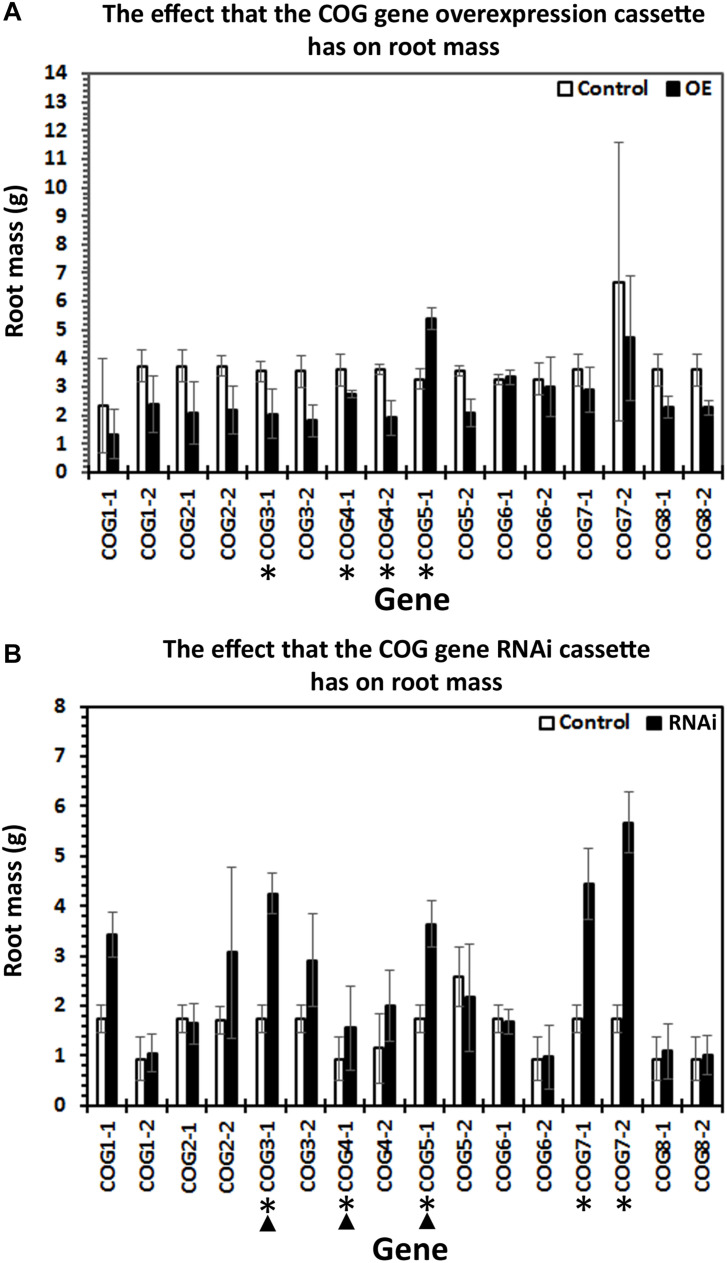
The effect the expression of the conserved oligomeric Golgi (COG) genetic cassette has on root mass. One-month-old transgenic root systems were weighed for their wet mass in grams (g) with three biological replicates with each biological replicate having between 10 and 25 root systems. Standard error is shown. (^∗^) Roots having statistically significant differences occurring between the control and COG-overexpressing (OE) or RNAi lines are indicated [*p* < 0.05, Mann–Whitney–Wilcoxon (MWW)]. (▲) Roots having statistically significant differences occurring between the control and COG-OE and the RNAi lines (*p* < 0.05, MWW). Experiments and statistical analyses have been performed from three independent biological replicates each having 10–25 independent transgenic root systems.

Differences occurring between the wr and pg analyses can be accounted for by root growth (Figure 4). As part of our analysis procedure, we enumerate root mass from fresh wet weight, allowing us to determine whether the expression of the COG gene cassette is influencing root growth. In these analyses, for a transgenic line to exhibit a statistically significant difference in root mass as compared to its respective control, all three biological replicates, each having between 10 and 25 experimental replicates (Supplementary Table 2), must be significantly different (*p* < 0.05, MWW). In the COG overexpression root mass analyses, COG3-1-OE, COG4-1-OE, and COG4-2-OE roots have a statistically significant decrease in root mass as compared to the pRAP15-*ccd*B control (*p* < 0.05, MWW). Conversely, COG5-1-OE root mass is statistically increased in comparison to the pRAP15-*ccd*B control (*p* < 0.05, MWW). In the RNAi transgenic lines, the COG3-1-RNAi, COG 4-1-RNAi, COG 5-1-RNAi, COG 7-1-RNAi, and COG 7-2-RNAi lines have a statistically significant increase in mass as compared to the pRAP17-*ccd*B control (*p* < 0.05, MWW). Among the studied COG genes, transgenic COG3-1 and COG4-1 roots are statistically significantly decreased in mass in the overexpression lines and increased in the RNAi lines (*p* < 0.05, MWW). In contrast, transgenic COG5-1 roots are statistically significantly increased in mass in both the overexpression and RNAi lines as compared to their pRAP15-*ccd*B and pRAP17-*ccd*B controls, respectively (*p* < 0.05, MWW). Consequently, by our analysis methods, COG3-1 and COG4-1 have an influence on *G. max* root growth.

### The Harpin Elicitor Influences the Expression of Conserved Oligomeric Golgi Genes

**FIGURE 5:**
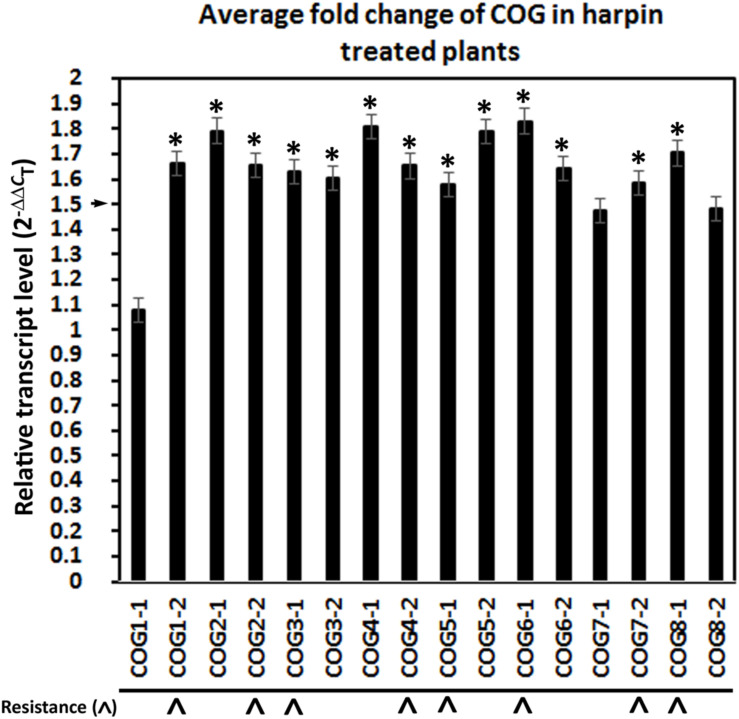
Treatment of *G. max* seeds with the bacterial effector harpin α/β results in the induction of conserved oligomeric Golgi (COG) gene expression. Gene expression calculated using the (2^–ΔΔ^*^*C*^*_*T*_) method (Livak and Schmittgen, 2001). (*) The gene is considered induced in expression if the relative level of expression is induced by 1.5-fold (p ≤ 0.05, Student’s *t*-test). (^∧^) COG genes having a function in resistance by the criteria set and demonstrated in the genetic engineering studies. RT-qPCR performed as already described.

Prior RT-qPCR-based experiments demonstrated that *G. max* seeds treated with a harpin α/β elicitor, in addition to suppressing *H. glycines* parasitism, leads to an increase in the relative transcript abundance of several resistance genes including α-SNAP (Aljaafri et al., 2017). The α-SNAP gene, located on chromosome 18, is a component of the *rhg1* locus and generally believed to be at least among the genes responsible for the *rhg1* phenotype (Matsye et al., 2011, 2012; Cook et al., 2012; Sharma et al., 2016). The α-SNAP gene has first been identified in *S. cerevisiae* as Sec17p (α-SNAP) (Novick et al., 1980). α-SNAP binds the syntaxin, Sed5p, which is involved in retrograde trafficking through its interaction with the COG complex (Shestakova et al., 2007). Therefore, an indirect link exists between COG and α-SNAP through Sed5p. In experiments presented here, RT-qPCR of cDNA template synthesized from RNA isolated from roots growing from harpin α/β-treated germinating seedlings results in an increase in the relative transcript abundances of >1.5-fold (*p* < 0.05, Student’s *t*-test) for 13 COG genes, including COG1-2, COG2-1, COG2-2, COG3-1, COG3-2, COG4-1, COG4-2, COG5-1, COG5-2, COG6-1, COG6-2, COG7-2, COG8-1 (Figure 5) (Aljaafri et al., 2017). The results are consistent with those obtained for other genes functioning in *G. max* resistance to *H. glycines* whose relative transcript abundances have been examined from harpin α/β-treated seedlings (Aljaafri et al., 2017; Lawaju et al., 2018; McNeece et al., 2019).

### Altered Conserved Oligomeric Golgi Gene Expression Influences Syntaxin 31 (SYP38) Transcript Abundance

**FIGURE 6:**
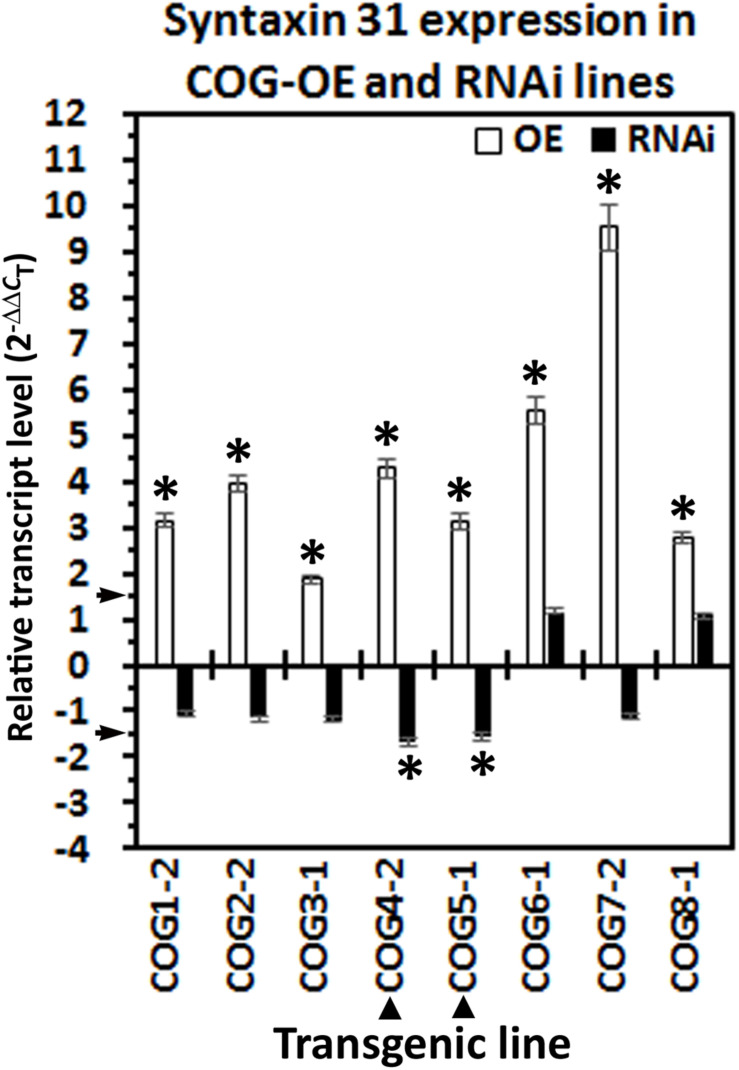
Syntaxin 31 expression in the transgenic conserved oligomeric Golgi (COG) lines. The cDNA made from the transgenic COG lines has been used in RT-qPCR experiments to determine the relative level of expression of syntaxin 31 (SYP38). (^∗^) Increased or decreased in its relative level of expression calculated using (2^–ΔΔ^*^*C*^*_*T*_) (Livak and Schmittgen, 2001). (▲) Syntaxin 31 is increased in its relative transcript abundance in the transgenic COG-overexpressing (OE) lines and decreased in its relative transcript abundance in the transgenic COG-RNAi lines. RT-qPCR performed as already described.

Prior experiments performed on *G. max* have shown that α-SNAP and syntaxin 31 (SYP38) (Glyma.14G064300) gene expression are co-regulated (Pant et al., 2014; Sharma et al., 2016). Analyses presented here have been performed to determine whether the relative transcript abundance of the *G. max* Sed5p homolog, syntaxin 31, is influenced by COG overexpression or RNAi. The results of those experiments are presented (Figure 6). In general, COG overexpression increases the relative transcript abundance of syntaxin 31 using RPS21 as a gene expression control (*p* < 0.05, Student’s *t*-test). Among these COG transgenic lines, however, the relative transcript abundance of syntaxin 31 is increased in the COG overexpression lines while also decreased in the COG RNAi lines only for COG4-2 and COG5-1 OE and RNAi transgenic lines (Figure 6). Therefore, COG4-2 and COG5-1 appear to influence the relative transcript abundance of syntaxin 31 at some level.

## Discussion

The *G. max* COG complex has been functionally characterized as it relates to its involvement in resistance to parasitism by *H. glycines*. The experiments have been performed because components of the vesicle trafficking machinery (myosin XI), vesicle tethering (Sec4), exocyst, and the membrane fusion complex (20S particle including SNARE) function in resistance to *H. glycines* (Cook et al., 2012; Matsye et al., 2012; Pant et al., 2014; Sharma et al., 2016, 2020; Klink et al., 2017). Among these genes is α-SNAP, a component of the “resistance to *heterodera glycines*” (*rhg1*) major *H. glycines* resistance locus (Matsye et al., 2011, 2012; Cook et al., 2012; Sharma et al., 2016). The observations are important from the standpoint that within these analyses occurred the identification of the *G. max* syntaxin 31 (SYP38), also functioning effectively in resistance (Pant et al., 2014). Syntaxin 31 is homologous to the *S. cerevisiae* Sed5p, a component of the SNARE complex functioning at the Golgi apparatus. In relation to the functional analysis presented here, Sed5p interacts with COG4 and COG6, which provides additional support to the hypothesis that the COG complex functions in the defense response that *G. max* has to *H. glycines* (Shestakova et al., 2007). Furthermore, Sed5p binds another protein, Sec17p (Hardwick and Pelham, 1992; Lupashin et al., 1997; Bubeck et al., 2008). Sec17 is the founding member of the α-SNAP gene family later identified in humans and then other organisms including plants (Novick et al., 1980; Clary et al., 1990; Sanderfoot et al., 2000). The α-SNAP gene is generally believed to be at least among the genes functioning at *rhg1* during resistance to *H. glycines* parasitism (Matsye et al., 2011, 2012; Cook et al., 2012, 2014; Bekal et al., 2015; Bayless et al., 2016, 2018; Sharma et al., 2016; Lakhssassi et al., 2017; Butler et al., 2019). However, its role within the complex, expressed *rhg1* locus containing various copies of three genes including α-SNAP, an amino acid transporter, and a wound inducible protein, copia retrotransposon, and various other structural elements are under continued study, which is important due to the nature of the locus (Melito et al., 2010; Cook et al., 2012, 2014; Bayless et al., 2016, 2018; Lakhssassi et al., 2017; Butler et al., 2019). Within this study, the principal findings are that the *G. max* COG complex families each has two members, one component of each gene family functions in defense, the expression of some of the family members are inducible by the bacterial effector harpin and some appear to influence the expression of syntaxin 31. These findings are discussed.

### The Conserved Oligomeric Golgi Complex Function

Original experiments that have been performed in *S. cerevisiae* led to the identification of the COG complex (Wuestehube et al., 1996; VanRheenen et al., 1998; Whyte and Munro, 2001; Ram et al., 2002). These findings were important to the analysis presented here because it has led to an understanding of how a number of components acting in vesicle transport may function in resistance in the *G. max*–*H. glycines* pathosystem. The COG complex functions in endosome-to-*trans* Golgi network (TGN) retrograde transport and is present in most eukaryotes (Koumandou et al., 2007). Therefore, it is expected to be involved in many cellular physiological processes, including protein stability, protein folding, protein–protein interactions, glycan-dependent quality control processes in the ER, among others (Moremen et al., 2012; Hebert et al., 2014). Consistent with this idea is the demonstration that, in *A. thaliana*, more than a thousand different *N*-glycosylated proteins have been identified with high confidence (Zielinska et al., 2010, 2012; Song et al., 2013; Strasser, 2016). As expected, mutations in COG genes generally lead to defects in glycosylation (Pokrovskaya et al., 2011). This defect occurs because of the impairment of recycling of enzymes functioning in glycosylation that would normally occur between the Golgi apparatus cisternae (Pokrovskaya et al., 2011). In *S. cerevisiae*, the COG complex interacts with the syntaxin Sed5p through its interactions with COG4 and COG6 (Shestakova et al., 2007). These observations mean that Sed5p interacts with both COG sub-complexes, indicating an important interaction that is central to cellular function. Observations also made in *S. cerevisiae* show that Sed5p interacts with Sec17p, the yeast homolog of α-SNAP (Hardwick and Pelham, 1992; Lupashin et al., 1997). Consequently, it could be expected that the COG complex would also perform a role in the resistant reaction that *G. max* has to *H. glycines*. The analysis presented here has been done because no functional analyses have been performed on COG complex genes as it relates to plant parasitic nematodes.

### The *G. max* Conserved Oligomeric Golgi Complex

The analysis presented here has identified the *G. max* COG complex genes. There are two *G. max* COG paralogs for each *A. thaliana* COG gene. This observation is consistent with the allotetraploid nature of the *G. max* genome (Schmutz et al., 2010). However, no localized gene copy number amplification has been observed for any of the COG complex gene families like what has been observed for another vesicle transport complex, the exocyst (EXOC) (Cvrčková et al., 2012). As already described, localized gene duplication has been reported to be important to the resistant reaction that *G. max* has to *H. glycines* (Cook et al., 2012, 2014; Lakhssassi et al., 2017). Like the COG complex, the plant exocyst is also composed of eight subunits (EXOC1–8), but the plant EXOC7 gene has experienced an extensive proliferation into many subtypes that likely have specialized functions (Cvrčková et al., 2012). The structure of the COG complex found in *G. max*, therefore, is also similar to that found for *S. cerevisiae* where its COG genes have been identified in four genetic screens (Wuestehube et al., 1996; VanRheenen et al., 1998; Whyte and Munro, 2001; Ram et al., 2002). Analyses have shown that while it is clear that COG components exist in plants, their level of conservation is low, with comparative analyses to human COG protein sequences occurring in the range of 20–34% (Latijnhouwers et al., 2005). The *G. max* COG complex protein sequence homology to its homologs in *A. thaliana* spans from a low of 62% between *A. thaliana* COG1 and *G. max* COG1-1 to a high of 82% occurring between the *A. thaliana* COG3 and *G. max* COG3-1 and *A. thaliana* COG8 and *G. max* COG8-2.

### Functional Analysis of *G. max* Conserved Oligomeric Golgi Complex Genes Root Growth

A functional analysis of three biological replicates, each having 10–25 experimental replicates, for the eight COG complex gene families has been performed for all 16 of its corresponding genes. The combination of impairing the susceptible reaction through COG gene overexpression in the otherwise *H. glycines*-susceptible *G. max*_[Williams 82/PI 518671]_ and facilitating parasitism in the otherwise *H. glycines*-resistant (*G. max*_[Peking/PI 548402]_) is taken as evidence supporting that the COG gene performs some role in the resistant reaction (Pant et al., 2014). The functional experiments revealed by RT-qPCR of each targeted gene demonstrate that it is possible to retrieve transgenic roots for each COG complex gene. Earlier studies have indicated that, in some cases, it is not possible to obtain transgenic roots for genes targeted for overexpression (myosin XI) possibly due to toxic effects caused by the transgenic cassettes (Austin et al., 2019). The toxic nature of even specific variant components of the *rhg1* locus, itself, and their direct binding partners has been reported (Bayless et al., 2016, 2018). These observations are consistent with the observation of the co-regulated nature of genes functioning in resistance in this pathosystem (Pant et al., 2014; Sharma et al., 2016; McNeece et al., 2019).

Part of the functional experiments used to determine the effect that the targeted resistance gene (COG complex component) has on *H. glycines* parasitism is to weigh the wet mass of each root. COG3-1-OE, COG4-1-OE, and COG5-1-OE roots have a 40.96, 23.68, and 46.8% decrease in root mass, respectively. In contrast, COG5-1 roots have a 1.64-fold increase in root mass. Complementary experiments using RNAi have also identified altered root mass in certain cases. For example, RNAi roots for COG3-1, COG4-1, COG5-1, COG7-1, and COG7-2 have increased masses of 2.45-fold, 1.66-fold, 2.09-fold, 2.56–fold, and 3.26-fold, respectively. The rest of the transgenic lines show no statistically significant effects in root mass regarding COG gene overexpression or RNAi. In comparisons of the transgenic experiments on the COG genes, COG3-1, COG4-1, and COG5-1 show effects in both the overexpression and RNAi lines. COG3-1 and COG4-1 show contrasting effects where their overexpression decreases root mass, while the RNAi increases root mass. COG5-1 shows an increase in both overexpression and RNAi lines. As has been reported for the *G. max* vesicle transport genes functioning in resistance to *H. glycines* parasitism, there appears to be a balance that is important to the function of the structure, and the balance in some cases appears to be manifested through co-regulated transcription and in its absence may be cytotoxic (Pant et al., 2014; Sharma et al., 2016; Bayless et al., 2016, 2018; Austin et al., 2019). Similar observations have been made in *A. thaliana* for COG7 *embryo yellow* (*EYE*) gene that functions in maintenance of the meristem (Ishikawa et al., 2008). The *eye* mutants are bushy, have shoot apical meristems with aberrant organization, and have an altered composition of their cell walls (Ishikawa et al., 2008). This is an important observation because the secreted hemicellulose-modifying gene xyloglucan endotransglycosylase/hydrolase (XTH) of *G. max* has been shown to have a significant role in the resistant reaction to *H. glycines* (Pant et al., 2014). Even the heterologous expression of the *G. max* XTH43 gene in *Gossypium hirsutum* (cotton) has led to a high degree of resistance to a different parasitic nematode, *Meloidogyne incognita* (Niraula et al., 2020). XTH is targeted to the Golgi apparatus prior to its secretion into the apoplast where it functions in cell wall modification (Fry, 1989; Fry et al., 1992; Nishitani and Tominaga, 1992; Nishitani, 1998). The Golgi apparatus, thus, serves prominently in processes involving cell wall modification, requiring the import of enzymes and glycoproteins from the ER to the Golgi *via* transition vesicles (Zhao and Colley, 2008; Handford et al., 2012). However, the synthesis of xyloglucan and modification of xyloglucan, itself, occur in the Golgi apparatus, first in the cisternae then moving to the medial- and trans-Golgi as XyG matures (Cocuron et al., 2007; Chevalier et al., 2010). Transport of the matrix polysaccharides and enzymes to the cell membrane then occurs through secretory vesicles (Kim and Brandizzi, 2016). The COG complex is also involved in homeostasis, pollen tube growth, and basic aspects of cell wall metabolism, so impairing individual components of the structure would be expected to have observable effects (Oda et al., 2015; Tan et al., 2016; Vukašinović et al., 2017; Rui et al., 2020).

### *G. max* Conserved Oligomeric Golgi Genes and a Role in the Resistance to *H. glycines* Parasitism

The results of the functional studies demonstrate that COG complex gene overexpression has the general capability to impair the ability of *H. glycines* to parasitize *G. max* roots, except for COG6-2 and COG7-2. The overexpression of the remaining *G. max* COG complex genes in analyses of *H. glycines* cysts in the wr system analyses leads to a low 62.68% decrease in *H. glycines* parasitism in the COG4-2-OE transgenic lines to a high 87.12% decrease in parasitism in the COG1-2-OE transgenic lines. In contrast, the RNAi of the COG complex genes has less of a general ability to affect *H. glycines* parasitism, occurring for nine of the 16 studied COG genes. The COG complex genes shown in RNAi experiments to function in resistance span a low 3-fold increase in *H. glycines* parasitism in COG8-1 to a high 22.1-fold in COG7-2. In all, through the combination of overexpression and RNAi, one member of each COG gene family has been shown to function in the resistance. The genes include COG1-2, COG2-2, COG3-1, COG4-2, COG5-1, COG6-1, COG7-2, and COG8-1. The demonstration that COG3-1 functions in the resistant reaction is consistent with the observations that the *hv*COG3 functions in resistance to fungal penetration in wheat (Ostertag et al., 2013). The conclusion drawn from the analysis presented here is that at least one gene from each COG complex gene family appears to function in defense. Furthermore, there appears to be a level of specificity regarding the genes that do participate in the resistant reaction since the remaining paralogs do not perform a role in defense. The results indicate that each component is important to the process, and it is possible that, by removing even just one component, the function of the whole structure is impaired. If this concept is true, the COG complex functions in a manner that is analogous to the exocyst by requiring each of its component parts for function. Furthermore, from the experiments presented here and results presented in other systems, we expect *G. max* COG genes to be expressed during the resistant reaction (TerBush et al., 1996; VanRheenen et al., 1998; Whyte and Munro, 2001; Ram et al., 2002; Kulich et al., 2010, 2015, 2018; Ostertag et al., 2013).

### How the Conserved Oligomeric Golgi Complex Results Relate to Defense in the *G. max*–*H. glycines* Pathosystem

Prior work in the *G. max*–*H. glycines* pathosystem accomplished the transcriptional mapping of the major resistance locus, *rhg1* (Matsye et al., 2011). Subsequent experiments have identified a number of variants within the locus and complex interactions occurring with their direct binding partners and even *H. glycines* effectors that may impair the function of these proteins (Cook et al., 2012; Bekal et al., 2015; Bayless et al., 2016, 2018; Lakhssassi et al., 2017). Subsequent functional studies demonstrated that the overexpression of α-SNAP in the *H. glycines*-susceptible genotype *G. max*_[Williams 82/PI 518671]_ leads to those susceptible plants exhibiting an approximately 60% decrease in parasitism (Matsye et al., 2012; Sharma et al., 2016). The results of those analyses have implicated that a functional vesicle transport apparatus plays an important defense role in *the G. max–H. glycines* pathosystem (Matsye et al., 2012; Pant et al., 2014; Sharma et al., 2016).

The α-SNAP gene has been first identified as the *Secretion* (*Sec*) gene (*Sec17*) in a mutant screen aimed to understand secretion (Novick et al., 1980, 1981). The α-SNAP gene name had been later denoted in studies of human cells and found to be homologous to *Sec17* (Clary et al., 1990). The α-SNAP protein interacts with syntaxin 31, mediating the delivery of transition vesicles to the *cis* face of the Golgi apparatus (Hardwick and Pelham, 1992; Lupashin et al., 1997; Bubeck et al., 2008; Matsye et al., 2012; Pant et al., 2014). Subsequent work has demonstrated the involvement of α-SNAP at all sites where vesicle and target membrane fusion occur, making it a universal component of membrane fusion (Zick et al., 2015). The importance of a functional 20S particle to pathogenesis has been revealed in a number of studies that have identified microbial neurotoxins that target SNARE proteins in animal systems and thus inhibit secretion (Schiavo et al., 1992a,b, 1994; Pellegrini et al., 1995; Chai et al., 2006; Jin et al., 2006; Strotmeier et al., 2012; Bennett et al., 2013). The COG complex relates to this structure because it interacts with a protein known as golgin-84 (Bascom et al., 1999; Sohda et al., 2010). Golgin-84 is a coiled-coil, integral membrane protein of the Golgi apparatus (Bascom et al., 1999). The interaction between the COG complex and golgin-84 is required for the assembly of a SNARE complex that functions in intra-Golgi retrograde transport (Sohda et al., 2010). Other interactions occurring between the COG complex and SNARE have also been observed, further strengthening the hypothesis that the COG complex performs an important function in intramembrane vesicle movement through specific target and vesicle membrane protein interactions (Suvorova et al., 2002; Shestakova et al., 2007; Laufman et al., 2011). The specific interactions include how the COG complex interacts directly with Syntaxin 6 as it positively regulates the endosome-TGN to facilitate retrograde transport (Laufman et al., 2011). Another interaction includes the COG complex and its interactions with Golgi SNAREs to facilitate intra-Golgi trafficking (Suvorova et al., 2002). The COG complex also interacts with syntaxin 5a, also known as Sed5p, to enhance intra-Golgi stability of the SNARE complex (Shestakova et al., 2007). Notably, Sed5p binds specifically to α-SNAP (Sec17p) at the *cis* face of the Golgi apparatus to facilitate vesicle fusion that ultimately leads to secretion (Hardwick and Pelham, 1992). Furthermore, other bacterial effectors that target the COG complex directly alter the movement of membrane to facilitate pathogen biology (Miller et al., 2017). The results presented here provide more context for those observations and show that by increasing the amount of individual COG complex gene expression, it is possible to facilitate an effective defense response. This process may happen because the other vesicle transport proteins are already being induced in their expression to accommodate a resistance reaction. In contrast, by impairing COG complex transcription, a normally *H. glycines*-resistant genotype like *G. max*_[Peking/PI 548402]_ facilitates parasitism.

### Components of the Conserved Oligomeric Golgi Complex Are Inducible by the Harpin Elicitor

The expression of the *G. max* COG genes can be induced by the bacterial elicitor harpin.

The harpin protein has been first identified from the causative agent of fire blight disease, *Erwinia amylovora*, which infects species of the Rosaceae (Wei et al., 1992). Harpins are heat-stable, glycine-rich proteins found in several gram-negative plant pathogenic bacteria and are secreted by the bacterial type III secretion system (Wei and Beer, 1993; Bogdanove et al., 1996; Choi et al., 2013). Harpins can function during the hypersensitive response but may also function through a systemic defense response (Wei et al., 1992; Dong et al., 1999; Neyt and Cornelis, 1999; Aljaafri et al., 2017). Harpin is known to function through ETI (Jones and Dangl, 2006). These interactions then lead to the transduction of signal through the mitogen-activated protein kinase (MAPK) cascade to elicit a defense response (Mindrinos et al., 1994; Grant et al., 1995; van der Biezen and Jones, 1998; Desikan et al., 1999, 2001; Lee et al., 2001; Mackey et al., 2002, 2003; Coppinger et al., 2004). Harpin, NDR1, and MAPKs have all been shown to be capable of functioning in the resistant reaction that *G. max* has to *H. glycines* and lead to the induced expression of α-SNAP, among other resistance genes (Aljaafri et al., 2017; McNeece et al., 2017, 2019). The genes also function to impair the pathogenicity of other pathogens of *G. max* (Lawaju et al., 2018). The results presented here showing the harpin-induced expression of COG genes functioning in resistance are consistent with observations that harpin can be effective in eliciting a resistant reaction (Aljaafri et al., 2017). However, observations have been made that show harpin can be deactivated through the activities of other bacterial effectors that specifically target it (O’Neill et al., 2018).

### Syntaxin 31 Transcript Levels Correlate to Conserved Oligomeric Golgi Gene Expression

Prior experiments have demonstrated the co-regulated nature of various *G. max* vesicle transport components involved in the resistant reaction to *H. glycines* (Pant et al., 2014; Sharma et al., 2016). The demonstration that Sed5p binds to Sec17p, COG4, and COG6 led to experiments to examine the relative transcript abundance of syntaxin 31 in the transgenic COG-overexpressing and RNAi lines (Hardwick and Pelham, 1992; Lupashin et al., 1997; Shestakova et al., 2007; Bubeck et al., 2008). The results of those experiments have revealed that there is an increased transcript level of syntaxin 31 in each of the transgenic COG-overexpressing lines that have been shown to impair *H. glycines* parasitism. These observations are consistent with experiments showing that several of the vesicle transport genes are co-regulated or that a balanced level of expression is important (Pant et al., 2014; Sharma et al., 2016; Bayless et al., 2016, 2018). However, the reciprocal experiments determining COG gene expression in syntaxin 31-overexpressing roots have not been performed. In contrast, suppressed syntaxin 31 expression is observed only in the COG4-2-RNAi and COG5-1-RNAi lines. In the other COG-RNAi lines, there is no observed effect on syntaxin 31 expression. This result, in itself, is interesting since it is these *G. max*_[Peking/PI 548402]_ transgenic lines that become susceptible to *H. glycines* parasitism. From these experiments, only the transgenic COG4-2 and COG5-1 are experiencing modulated syntaxin 31 expression that correlates to the respective COG overexpression (induced syntaxin 31 transcription) or RNAi lines (suppressed syntaxin 31 transcription). Notably, COG4 and COG5 occur on each of the two lobes of the COG complex so signals relating to each lobe of the COG complex may be important in this regulated transcription. Furthermore, prior experiments have shown that Sed5p binds COG4p (Shestakova et al., 2007).

### Summary

Genes composing the *G. max* COG complex have been identified. Furthermore, transgenic functional analyses, a harpin elicitor study, and the effect that COG component overexpression/RNAi have on syntaxin 31 expression have been performed (Table 2). Furthermore, the combination of COG gene overexpression in the *H. glycines*-susceptible *G. max*_[Williams 82/PI 518671]_ (along with the appropriate pRAP15-*ccd*B control) and RNAi in the *H. glycines*-resistant *G. max*_[Peking/PI 548402]_ (along with the appropriate pRAP17-*ccd*B control) has led to the identification of eight COG genes that perform a role in resistance. An RT-qPCR analysis targeting each of the 16 *G. max* COG genes using RNA isolated from harpin α/β-treated seeds has resulted in the determination that 12 of them are increased in their relative transcript abundance. Lastly, altered COG expression affects the transcript abundance of syntaxin 31. The overall results are consistent with those observed in other protein complexes that relate to vesicle transport. Specifically, the exocyst requires each component for the assembly and function of the structure. Furthermore, the balanced expression, possibly through regulated co-expression of components relating to the resistance reaction that *G. max* has to *H. glycines*, is also receiving similar treatment. The further analyses of these and additional genes that relate to the vesicle transport process will undoubtedly identify further intricacies regarding their involvement during the resistant reaction. Some of these interactions may come from yet unexplored membrane components. For example, experiments have shown the COG complex protein COG2 with EXOC70A1 *via* vesicle tethering 1 (VETH1) and VETH2 to promote xylem vessel differentiation (Oda et al., 2015). These recent results indicate that the cross-talk that is occurring between these different components functioning in vesicle trafficking is more prevalent and more important than previously understood. Furthermore, the results indicate it is possible that *H. glycines* may specifically target other Golgi-associated proteins for its benefit like what it already does for *a*-SNAP.

**TABLE 2:** Summary of the data pertaining to this analysis of the *G. max* conserved oligomeric Golgi (COG) complex.

Gene	OE-R	RNAi-S	OE-R and RNAi-S	COG-OE: SYP38-I	COG-R: SYP38-D	Harpin-I
COG1-1	Yes	No	No	n/a	n/a	No
COG1-2*	Yes	Yes	Yes	Yes	No	Yes
COG2-1	Yes	No	No	n/a	n/a	Yes
COG2-2*	Yes	Yes	Yes	Yes	No	Yes
COG3-1*	Yes	Yes	Yes	Yes	No	Yes
COG3-2	Yes	No	No	n/a	n/a	Yes
COG4-1	Yes	No	No	n/a	n/a	Yes
COG4-2*	Yes	Yes	Yes	Yes	Yes	Yes
COG5-1*	Yes	Yes	Yes	Yes	Yes	Yes
COG5-2	Yes	No	No	n/a	n/a	Yes
COG6-1*	Yes	Yes	Yes	Yes	No	Yes
COG6-2	No	No	No	n/a	n/a	Yes
COG7-1	No	Yes	No	n/a	n/a	No
COG7-2	Yes	Yes	Yes	Yes	No	Yes
COG8-1*	Yes	Yes	Yes	Yes	No	Yes
COG8-2	Yes	No	No	n/a	n/a	No

OE-R, COG overexpression-resistant outcome; RNAi-S, COG RNAi-susceptible outcome; OE-R and RNAi-S, COG overexpression-resistant outcome and COG RNAi-susceptible outcome; COG-OE: SYP38-I, COG overexpression leads to syntaxin 38 increased expression; COG-RNAi: SYP38-S, COG RNAi leads to syntaxin 38 decreased expression; Harpin-I, Harpin treatment leads to an increase in targeted COG gene expression. *Functions in resistance.

## Data Availability Statement

The original contributions presented in the study are included in the article/Supplementary Material, further inquiries can be directed to the corresponding author.

## Author Contributions

BL: conceptual planning, identifying COG genes, comparative analyses to *A. thaliana* proteome, PCR primer design, cloning genes, plant genetic transformation, nematode infections, cyst extractions, qRT-PCR, quantitative analyses, and manuscript preparation. PN: affymetrix data extraction and analysis, qRT-PCR, and quantitative analyses. GL and KL: conceptual planning, nematode infections, cyst extractions, and quantitative analysis. VK: conceptual planning, quantitative analyses, and manuscript preparation. All authors contributed to the article and approved the submitted version.

## Conflict of Interest

The authors declare that the research was conducted in the absence of any commercial or financial relationships that could be construed as a potential conflict of interest.

## Footnotes

1 https://phytozome.jgi.doe.gov

## Supplementary Material

Supplementary Table 1 PCR and RT-PCR primers used in the analysis.

Supplementary Table 2 Number of experimental and biological replicates employed in the analysis.

## Abbreviations

COG: conserved oligomeric Golgi
ETI: effector triggered immunity.
